# Effectiveness of mat Pilates or equipment-based Pilates in patients with chronic non-specific low back pain: a protocol of a randomised controlled trial

**DOI:** 10.1186/1471-2474-14-16

**Published:** 2013-01-09

**Authors:** Maurício Antônio da Luz, Leonardo Oliveira Pena Costa, Fernanda Ferreira Fuhro, Ana Carolina Taccolini Manzoni, Naiane Teixeira Bastos de Oliveira, Cristina Maria Nunes Cabral

**Affiliations:** 1 Universidade Cidade de São Paulo, Rua Cesário Galeno 475, São Paulo, 03071-000, Brazil; 2 Musculoskeletal Division, The George Institute for Global Health, Sydney, Australia; 3 Physical Therapy Department, Universidade Cidade de São Paulo, São Paulo, Brazil

**Keywords:** Pilates-based exercises, Low back pain, Disability

## Abstract

**Background:**

Chronic low back pain is an expensive and difficult condition to treat. One of the interventions widely used by physiotherapists in the treatment of chronic non-specific low back pain is exercise therapy based upon the Pilates principles. Pilates exercises can be performed with or without specific equipment. These two types of Pilates exercises have never been compared on a high-quality randomised controlled trial.

**Methods/design:**

This randomised controlled trial with a blinded assessor will evaluate eighty six patients of both genders with chronic low back pain, aged between 18 and 60 years, from one Brazilian private physiotherapy clinic. The patients will be randomly allocated into two groups: Mat Group will perform the exercises on the ground while the Equipment-based Group will perform the Pilates method exercises on the following equipment: Cadillac, Reformer, Ladder Barrel, and Step Chair. The general and specific disability of the patient, kinesiophobia, pain intensity and global perceived effect will be evaluated by a blinded assessor before randomisation and at six weeks and six months after randomisation. In addition, the expectation of the participants and their confidence with the treatment will be evaluated before randomisation and after the first treatment session, respectively.

**Discussion:**

This will be the first study aiming to compare the effectiveness of Mat and Equipment-based Pilates exercises in patients with chronic non-specific low back pain. The results may help health-care professionals in clinical decision-making and could potentially reduce the treatment costs of this condition.

**Trial registration:**

Brazilian Registry of Clinical Trials RBR-7tyg5j

## Background

Chronic low back pain is a common musculoskeletal problem worldwide [1] with an unfavorable prognosis [2]. Back pain is the second most prevalent health condition in Brazil [3] and is associated with high costs [4]. The etiology of low back pain is still unknown, but it is believed to be multifactorial. Therefore, degenerative, mechanical and postural conditions can be associated with low back pain [5-7]. Exercise therapy has been considered by the current clinical practice guidelines [8,9] as an effective treatment for chronic low back pain.

Exercise based upon the Pilates principles have been extensively used as an intervention for patients with chronic low back pain [6-10]. These exercises can be performed with specific apparatus (Equipment-based Pilates) or without them (Mat Pilates) [11]. Eight basic principles are considered to be essential while performing this intervention: diaphragmatic breathing, control, concentration, centering, precision, flowing movements, strength, and relaxation [12]. Previous systematic reviews on the effectiveness of the Pilates exercises in patients with low back pain revealed conflicting results [13-15]. While one review concluded that Pilates are effective for pain [13], other concluded that Pilates are effective for pain and disability [15] and finally the most recent review concluded that Pilates exercises were not effective in the reduction of pain and disability [14]. However, the authors from these reviews stated that most of the available trials are typically small and with suboptimal quality [13-15]. These issues reinforce the need for more studies on the effectiveness of Pilates exercises in the treatment of patients with chronic low back pain. To our knowledge there is no study that compared the effects of Mat-Pilates against Equipment-based Pilates in patients with chronic low back pain. The objective of this randomised controlled trial will be to compare the effectiveness of Mat Pilates and Equipment-based Pilates in the treatment of chronic non-specific low back pain patients. This study is likely to provide important information on the effectiveness and costs of these treatments as Equipment-based Pilates is more expensive than Mat Pilates for both physiotherapists and patients.

## Methods/design

### Study design

This is a randomised controlled trial with a blinded assessor that aims to assess the effectiveness of Mat and Equipment-based Pilates exercises in patients with chronic non-specific low back pain. The study has been approved by the Research Ethics Committee of the Universidade Cidade de Sao Paulo (PP 13610106), and is funded by National Counsel of Technological and Scientific Development (CNPq), Brazil (479645/2011-6). All participants will sign a consent form to agree to participate in the study.

### Participants

Our final sample will be composed by 86 patients seeking care for chronic non-specific low back pain from a private outpatient physiotherapy department from the city of Campo Limpo Paulista, Brazil, with a duration of symptoms of at least three months and aged between 18 and 60 years. The contraindications for exercise will be assessed with the Physical Activity Readiness Questionnaire, which is recommended as the minimal standard for pre-participation assessment as it can identify through a positive response the participants who need prior medical evaluation and authorization [16]. The exclusion criteria will be regular Pilates practice, pregnancy, serious spinal pathologies (fractures, inflammatory disorders or tumours), serious neurological impairment, inability to speak or write in Portuguese, and physiotherapy treatment for non-specific chronic low back pain in the past six months [17,18].

### Sample calculation

A sample of 86 participants was determined by a sample size calculation designed to detect a clinically important difference of one point in the Pain Numerical Rating Scale (estimated standard deviation = 1.4 points), one point in the Patient-Specific Functional Scale (estimated standard deviation = 1.4 points), one point in the Global Perceived Effect Scale (estimated standard deviation = 1.3 points), and four points in the Roland-Morris Disability Questionnaire (estimated standard deviation = 4.9 points) across all time points. These between-group differences were chosen based upon minimally important differences for patients with low back pain [19]. The following specifications will be considered: α=0.05, statistical power of 80% and follow-up loss of up to 15%. A correlation of r=0.50 between the follow-up assessments within the outcome measurement was assumed in these calculations.

### Intervention

All participants will receive their treatments based upon the Pilates principles. In the first treatment session, they will be given basic instructions on the method and training to activate core strength, i.e. isometric contraction of the transversus abdominis, perineal, gluteal, and multifidus muscles at expiration during diaphragmatic breathing. This core strength activation is also known as “powerhouse” [5,11,12,17]. Sessions will last one hour and will be provided twice a week over a period of six weeks. All exercises will be modified so that they can be performed at three levels of difficulty: basic, intermediate, and advanced. Some basic exercises can be tailored for each patient. For example, the “hip opener” can be performed in a lower range of motion and without resistance if needed. Similarly, other exercises, such as the “roll up”, can be progressed by using additional resistance. In the cases where all adaptations where not useful, the exercise will be replaced by another with the same objective. The level of difficulty for each exercise will be determined according to individual needs and increased as the participants reduce postural compensation [17,20,21]. Additional file 1: Appendix 1 presents a complete description of both treatments.

### Procedures

Patients will be assessed by an outcome assessor who will be unaware of the patient's allocation. This assessor will determine the eligibility of the patients as well as will collect the outcomes of the study. Outcomes will be collected at baseline, six weeks and six months after randomisation. Due to the nature of the interventions it will not be possible to blind the participants as well as the physiotherapist.

**Figure 1:**
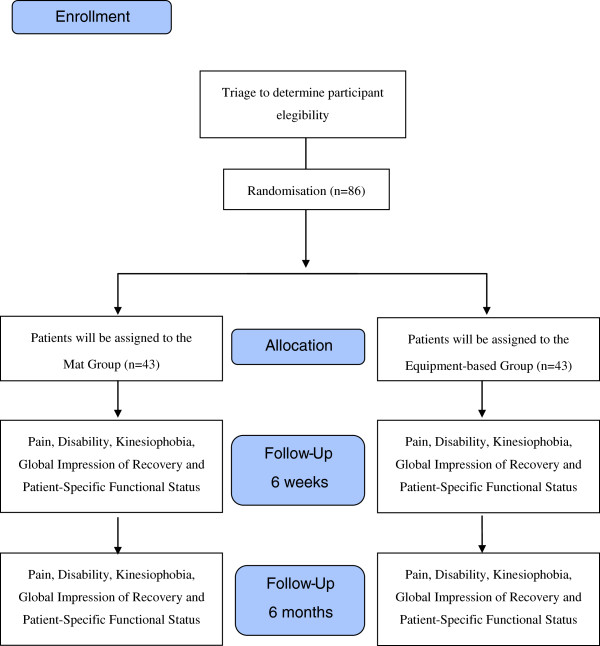
Flow diagram of the study.

A simple randomization schedule was performed using Microsoft Excel for Windows by an independent researcher who will not be involved in participant recruitment and assessments. After the initial assessment, the participants will be referred to the physiotherapist overseeing the intervention and will be randomly allocated to one of two groups using consecutively numbered sealed opaque envelopes. The envelopes will contain a sentence that will inform the participants of their group allocation: Mat Group or Equipment-based Group (Figure 1). The Mat Group will perform the exercises on the ground. The Equipment-based Group will perform the Pilates method exercises on the following equipment: Cadillac, Reformer, Ladder Barrel, and Step Chair.

This trial commenced recruitment in October 2011 and the last follow up is expected to be performed in November 2012.

### Outcome measures

#### Primary outcomes

Pain intensity will be measured by the Pain Numerical Rating Scale [22] which is an 11-point numerical scale (ranging from zero to 10), in which zero represents “no pain” and 10 represents “pain as bad as could be”. The participants will be asked to classify their average pain over the last week.

Disability associated with low back pain will be measured by the Roland-Morris Disability Questionnaire [23]. This questionnaire consists of 24 yes/no questions (total score ranging from zero to 24) related to normal activities of daily living in which every affirmative answer represents one point. The final score is the sum of these points. Lower scores represent less disability.

#### Secondary outcomes

Global Impression of Recovery will be measured by the Global Perceived Effect Scale [24]. This scale compares the onset of symptoms to the last few days. It is an 11-point scale (ranging from −5 to +5) in which −5 is “vastly worse”, zero is “no change”, and +5 is “completely recovered”. Higher score means greater recovery from the condition.

Function will be measured by the Patient-Specific Functional Scale [25]. The participants will identify three important activities in which they are having difficulty or which they are unable to perform due to chronic low back pain at the time of the assessment. Additionally, the participants will indicate on an 11-point scale (ranging from zero to 10) how capable they feel of performing these activities, with zero meaning “unable to perform the activity” and 10 meaning “able to perform the activity at pre-injury level”. The total score is calculated by the average of these three activities and ranges from zero to 10, with higher scores representing greater specific functional ability.

Fear of movement will be measured by the Tampa Scale for Kinesiophobia [26], which is a 17-item questionnaire with likert-type scales ranging from 1 “strongly disagree”, 2 “partially disagree”, 3 “partially agree” and 4 “strongly agree”. The final score ranges from 17 to 68 points, with higher scores indicating a higher degree of kinesiophobia.

All instruments described above have been translated and cross-culturally adapted into Brazilian-Portuguese and their measurement properties have been tested. The measurement properties of the Brazilian-Portuguese versions of these instruments are very similar to the original versions in English [27-31] with good estimates of reproducibility, construct validity and responsiveness.

We will be also measure patients’ expectancy and credibility with treatment. Expectancy will be measured by the Numerical Scale of Expectancy for Improvement. This is a method of assessing a patient’s expectation of improvement after treatment on a scale ranging from zero to 10, where zero represents “no expectancy for improvement” and 10 represents “expectancy for the greatest possible improvement”. This scale will only be used prior to the randomisation. The Treatment Credibility Scale is composed of four questions that assess the participants’ degree of confidence that if their symptoms will improve and confidence in the proposed treatment. The score ranges from zero to six, with zero being “not at all confident” and six being “very confident”. It will only be used after the first treatment session in both groups [32]. This scale is being translated and cross-culturally adapted into Brazilian Portuguese by our research group.

### Statistical analysis

Data will be double-entered. The statistical analysis will be performed on an intention-to-treat basis. The effects of the intervention on pain intensity, global impression of recovery, general and specific disability, and kinesiophobia will be calculated using linear mixed models [33], which will incorporate terms for treatment, time, and treatment by time interactions (these interaction terms are equivalent to the between-group differences adjusted for the baseline estimates). The level of significance will be set at α=0.05. The data will be analyzed using the SPSS 19 for Windows by a blinded statistician who will receive coded data.

## Discussion

Although the use of Pilates exercises in the treatment of chronic low back pain has steadily increased, the current evidence of its effectiveness is still supported by a low number of studies with suboptimal methodological quality [5,17,18,21,34-36], being only one study achieving a high score on the PEDro scale [18], this reinforces the need for new high quality studies [13-15].

Our study was designed with a number of features in order to achieve the best possible methodological quality. Therefore our study can provide a better understanding about possible differences in the treatment effectiveness of these two Pilates approaches in patients with chronic low back pain. The results of this study can help physiotherapists on their clinical decision-making as well could help to guide possible investments in physiotherapy clinics as the Equipment-based Pilates is more expensive than the Mat Pilates. To our knowledge, this is the largest randomised controlled trial that used Pilates interventions in patients with chronic low back pain and the first to compared Mat and Equipment-based Pilates in this population.

In this protocol, we demonstrated the basic principles and description of the design of a randomised controlled trial comparing the effectiveness of Mat Pilates and Equipment-based Pilates in patients with chronic non-specific low back pain. At the time of submission of this protocol, 86 participants had been randomised and completed the six-week follow-up and 78 completed the 6 months follow-up. To date, there has been only one loss of follow-up at six weeks and two losses of follow-up at six months. We estimate that the results will be published in the second semester of 2013.

## Competing interests

The authors declare that they have no competing interests.

## Author’s contributions

Authors MALJ, LOPC, FFF, ACTM, NTBO and CMNC have all contributed to conception and design of this trial, have been involved in drafting the manuscript and revised it critically and have given final approval of this version to be published.

## Pre-publication history

The pre-publication history for this paper can be accessed here:

http://www.biomedcentral.com/1471-2474/14/16/prepub

## Supplementary Material

Additional file 1 **Appendix 1.** Description of Pilates exercises in the treatment of low back pain. Click here for file
