# Supplementary material for: Effectiveness of mat Pilates or equipment-based Pilates in patients with chronic non-specific low back pain: a protocol of a randomised controlled trial
Source: BMC Musculoskelet Disord. 2013 Jan 9;14:16. doi: 10.1186/1471-2474-14-16 (PMC3544561; doi:10.1186/1471-2474-14-16)
Supplement: Additional file 1 — Appendix 1. Description of Pilates exercises in the treatment of low back pain. [file 1471-2474-14-16-S1.doc]

| **Equipment-based Pilates Exercises** | | |
| --- | --- | --- |
| **1. Barrel** | | |
| **Stretching the hamstrings** | Patient in standing position with one leg on the barrel.  Patient peels down the spine (in a C-curve).  Repeat 4-10 times.  Return to the starting position. | **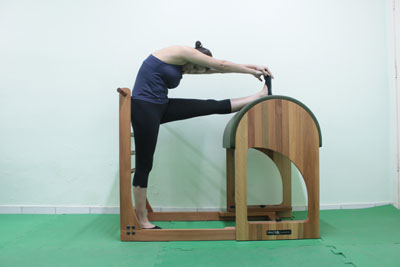** |
| **Stretching the adductor muscles** | Patient in standing position on the side of the barrel with one leg on the barrel.  Patient stretches up and over the body.  Repeat 4-10 times.  Return to the starting position. | 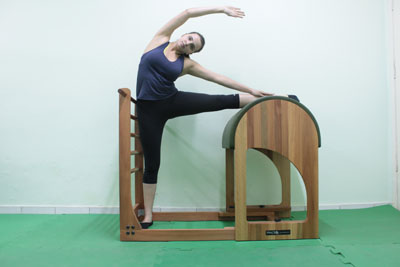 |
| **Stretching of gluteus muscle** | Patient in standing position with one leg on the barrel with his/her knee in 90 degrees of flexion with rotation and abduction of the hip.  Patient peels down the spine (in a C-curve).  Repeat 4-10 times.  Return to the starting position. | 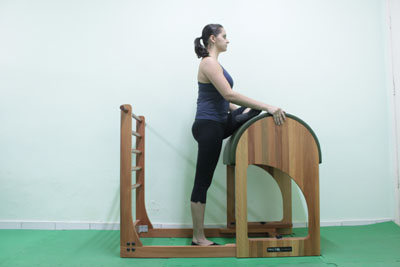 |
| **Spinal tilt** | Patient in lying position with the side of the body on the barrel.  The body should be aligned and relaxed.  The free arm should stretches up and over the body.  Repeat 4-10 times.  Return to the starting position. | 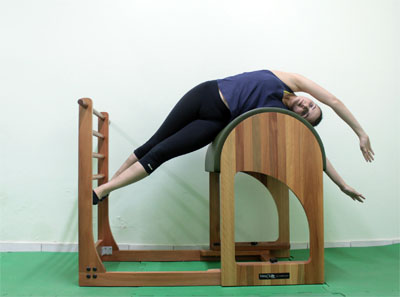 |
| **Stretching the back muscles** | Patient in lying position on the barrel, forehead down.  Your body aligned and relaxed.  The body should be aligned and relaxed.  The free arm should stretches up and over the body.  Return to the starting position. | 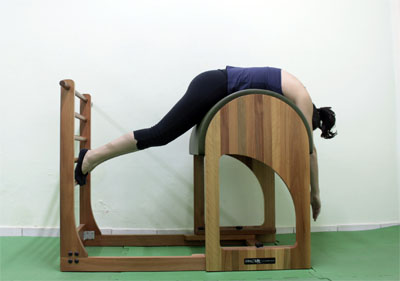 |
| **Strengthening the oblique muscles** | Patient sit up on his/her side on the barrel, with the feet resting on the step.  Patient activates the powerhouse and contracts the oblique muscles by bending the trunk sideways.  Repeat 4-10 times.  Return to the starting position. | 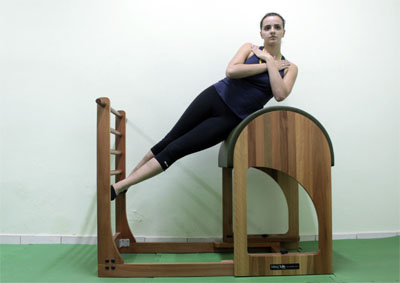 |
| **Strengthening of abdominal muscles** | Patient sits up on the barrel, with his/her feet resting on the step.  Patient activates the powerhouse while peeling down the spine.  Repeat 4-10 times.  Return to the starting position. | 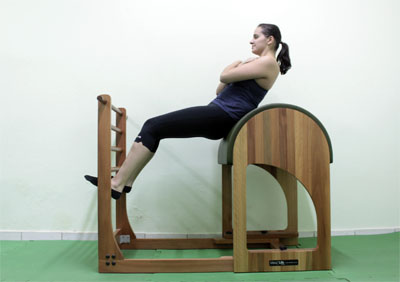 |
| **2. Step chair** | | |
| **Strengthening of abdominal muscles and stretching of back muscles** | Patient stands behind the step chair, with the body in C-Curve position with the hands on the step.  Press the step down.  Repeat 4-10 times.  Return to the starting position. | **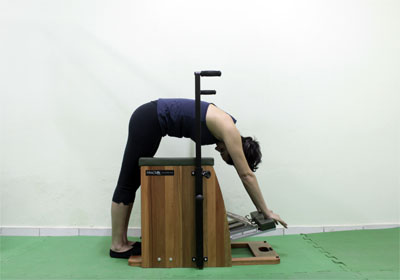** |
| **Strengthening of abdominal muscles** | Patient sits on the chair, with the body in C-Curve position, legs abducted and hands on the step.  Press the step down.  Repeat 4-10 times.  Return to the starting position | **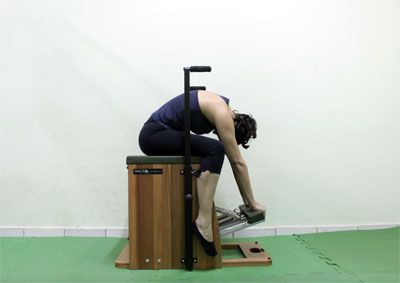** |
| **Strengthening of oblique muscles** | Patient sits on his/her side on the chair, with one hand on the step.  The free arm should stretch up and over the body.  Press the step down.  Repeat 4-10 times.  Return to the first position. | **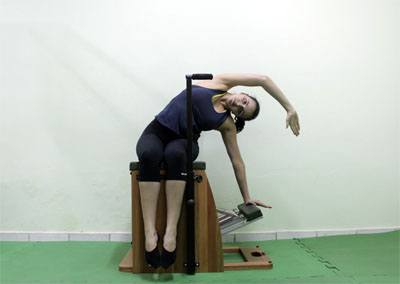** |
| **3. Cadillac** | | |
| **Push through round back** | Patient sits up tall with straight legs abducted and hands on the bar.  Then patient C-Curves from the base of his/her spine and pull the bar down, stretching the spine.  Retrograde the C-Curve, keeping the shoulders down.  Repeat 4-10 times.  Return to the starting position. | 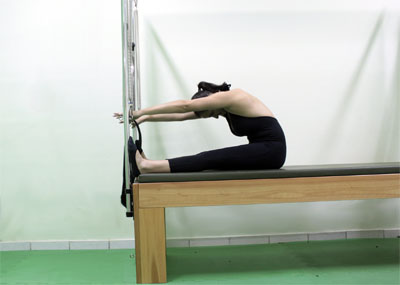 |
| **Side Stretch** | Patient sits on the side of the table, legs hanging off with one arm pushing the bar down.  The free arm should stretch up and over the body diagonally.  Repeat 4-10 times.  Return to the starting position. | 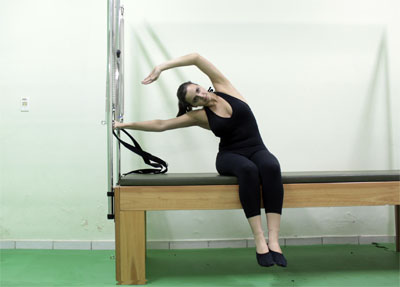 |
| **Port-de-Bras** | Patient sits up tall with both legs stretched in front of the Cadillac.  One hand holds the bar, while the other arm stretches the opposite diagonal side, stretching the oblique muscles.  Repeat 4-10 times.  Return to the starting position. | 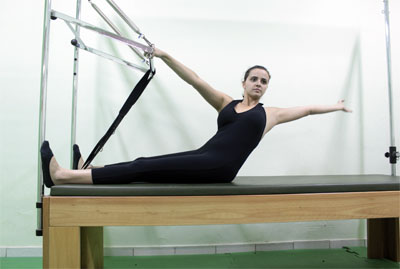 |
| **Hip Opener** | Patient in lying on his/her side with one foot on the bar and knees flexed.  Patient pushes the bar up while straightening both legs.  Repeat 4-10 times.  Return to the starting position. | 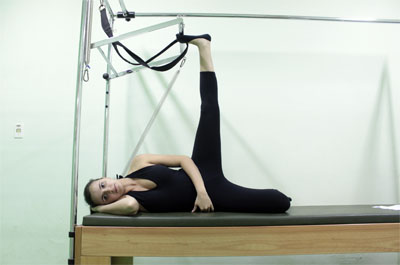 |
| **Roll Up** | Patient in supine position with his/her hands holding the bar.  Patient pushes the bar while making a the C-Curve.  Patient lifts the back off the table.  Repeat 4-10 times.  Return to the starting position. | 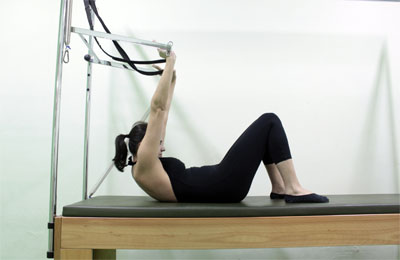 |
| **Bridge** | Patient lying supine with his/her legs in the loop of the Trapeze.  Patient activates the powerhouse, while lifts the pelvis off the table.  Repeat 4-10 times.  Return to the starting position. | 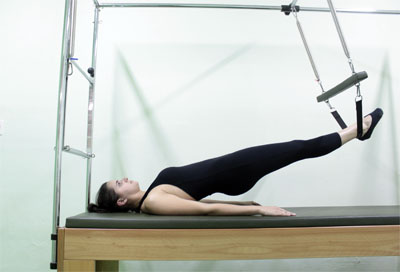 |
| **Stretching of back muscles** | Patient in standing position not facing the Cadillac with his/her hands holding the bars and feet supported on the foot base.  Patient lift the feet from the foot base stretching the back muscles.  Repeat 4-10 times.  Return to the starting position. | 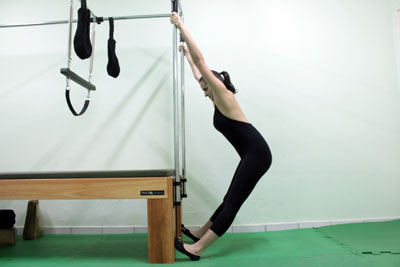 |
| **Cat kneeling** | Patient kneels down on the Cadillac with hands holding the bar.  Patient pushes the bar downwards and peels the spine down.  Patient then pushes the bar forward and extends the trunk.  Repeat 4-10 times.  Return to the starting position. | 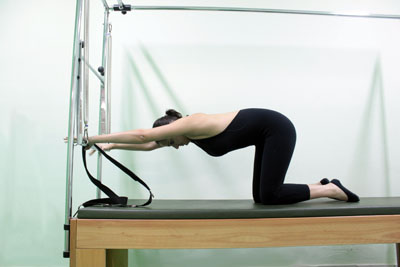 |
| **4. Reformer** | | |
| **Strengthening of the gluteus and stretching of the hamstrings** | Patient stands beside the Reformer, with both hands on the bar, one leg on the carriage and the other one on the floor.  The leg positioned on the carriage slides it.  Repeat 4-10 times.  Return to the starting position. | 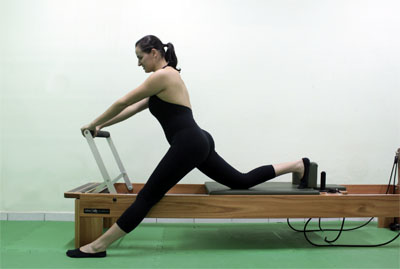 |
| **Elephant** | Patient in standing position on the carriage with his/her hands holding the bar, slides the carriage.  This exercise aims to stretch the back and lower limb muscles.  Repeat 4-10 times.  Return to the starting position. | 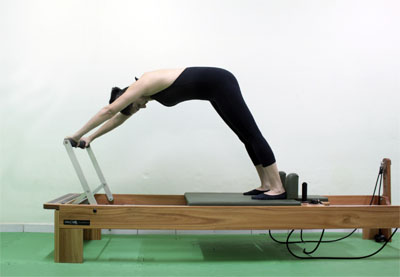 |
| **Stretching of hamstrings - C-curve** | Patient sits on the carriage with the hands holding the bar with the feet on the reformer’s base.  With the knees in 90 degrees, the patient slides the carriage making a C-curve and stretching the hamstrings.  Repeat 4-10 times.  Return to the starting position. | 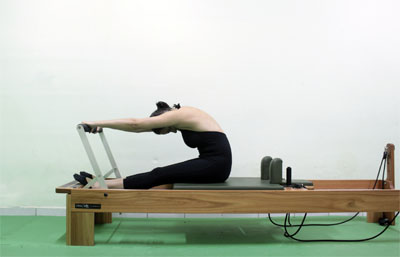 |
| **Knee stretch series: round back** | Patient kneels on the carriage with both hands holding the bar.  Patient then activates the powerhouse and slides the carriage.  Repeat 4-10 times.  Return to the starting position. | 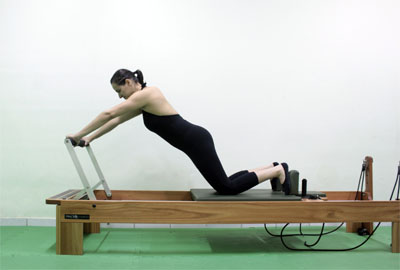 |
| **Stretching the spinal muscles** | Patient sits on his/her side on the carriage with one hand holding the bar.  The free arm should stretch up and over the body while sliding the carriage.  Repeat 4-10 times.  Return to the starting position. | 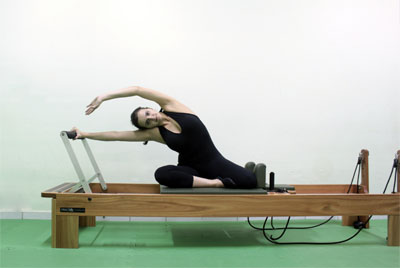 |
| **The hundred** | Patient in prone position with both arms straight holding the handles, both legs should be completely extended.  Patient activates the powerhouse while slides the carriage.  Emphasis should be done on the abdominal muscles.  Repeat 4-10 times.  Return to the starting position. | 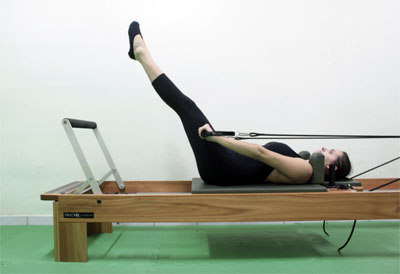 |
| **Running** | Patient in prone position on the carriage with both feet on the bar.  Patient simulates running by flexing and extending the knees.  Repeat 4-10 times.  Return to the starting position. | 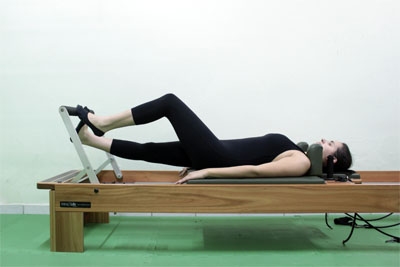 |
| **Mat Pilates exercises** | | |
| **Swan** | Patient in supine with the upper limbs along the body.  Patient extends the trunk using both arms, keeping the pelvis on the mat and arching the upper thoracic spine.  Repeat 4-10 times.  Return to the starting position. | 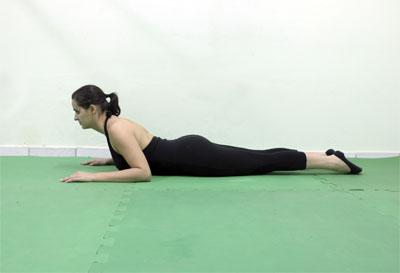 |
| **Child's Pose for Pilates** | Patient kneels and sits on his/her ankles.  Patient flexes the trunk towards the mat using his/her arms.  Repeat 4-10 times.  Return to the starting position. | 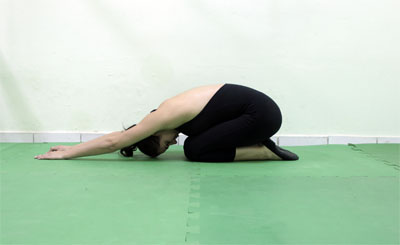 |
| **The modified hundred** | Patient in supine with knees flexed and both feet on the mat.  Patient moves the chin towards the chest making a C-curve and lifting the back from the mat.  Patient should maintain this position while moving the arms upwards and backwards using the powerhouse.  Repeat 4-10 times.  Return to the starting position. | 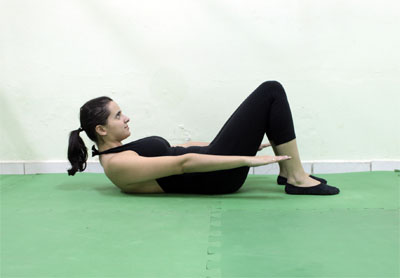 |
| **Roll Up** | Patient in supine with both legs straight keeping the body aligned. Patient’s upper limbs must be fully flexed.  Patient moves the chin towards the chest making a C-curve, lifting the back from the mat and moving both hands towards the feet.  Repeat 4-10 times.  Return to the starting position. | 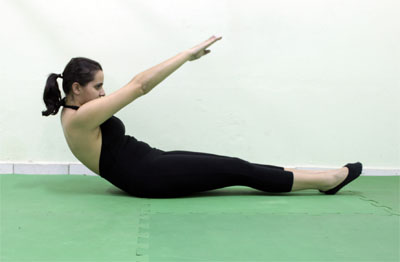 |
| **One Leg Circle** | Patient in supine with one leg supported on the mat while the other is flexed without support. Patient should move the leg simulating a circle figure.  Repeat 4-10 times.  Return to the starting position. | 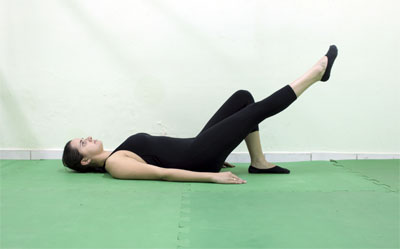 |
| **Spine Twist** | Patient sits with the body aligned with the arms along the body.  Patient moves the hand towards the contralateral foot.  Repeat 4-10 times.  Return to the starting position. | 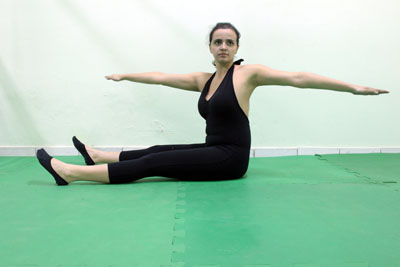 |
| **Rolling like a Ball** | Patient sits with knees and hips flexed with the hands holding the legs above the ankles.  Patient should lift both feet from the mat, rolling backwards.  Repeat 4-10 times.  Return to the starting position. | 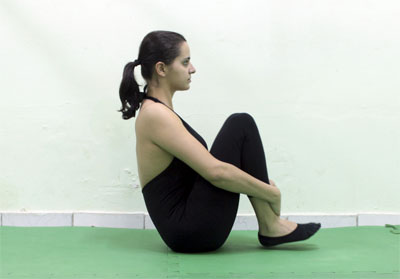 |
| **Single Leg Stretch** | Patient in supine with one leg flexed and one hand holding the one leg above the ankle. The other leg should be in 45 degrees of flexion.  Patient should keep the C-curve moving the back from the mat during the exercise, alternating each of the legs.  Repeat 4-10 times.  Return to the starting position. | 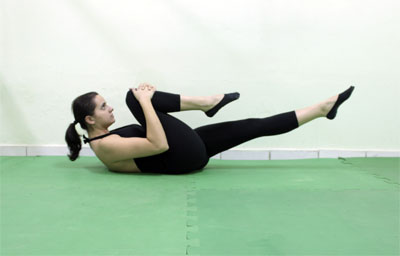 |
| **Chest Lift with Rotation** | Patient in supine with both knees flexed and with both hands positioned on the back of the neck.  Patient should flex and rotate the trunk, moving the elbow toward the contralateral knee.  Repeat 4-10 times.  Return to the starting position. | 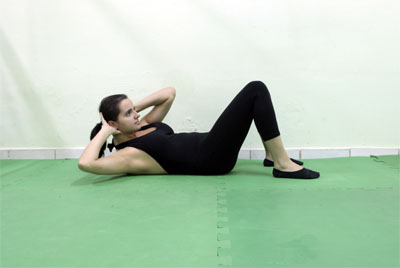 |
| **Double Leg Stretch** | Patient in supine with both legs straight keeping the body aligned.  Patient flexes the trunk, hips and knees, moving the forehead towards the knees, hugging both legs.  Repeat 4-10 times.  Return to the starting position. | 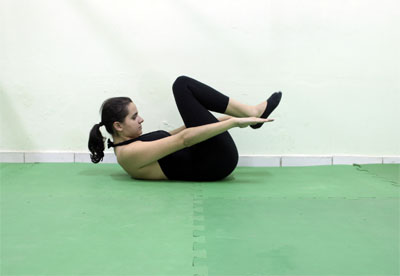 |
| **Shoulder Bridge** | Patient in supine with knees flexed and both feet on the mat.  Patient should keep the back and upper limbs on the mat, lifting the hips from the mat only.  Repeat 4-10 times.  Return to the starting position. | 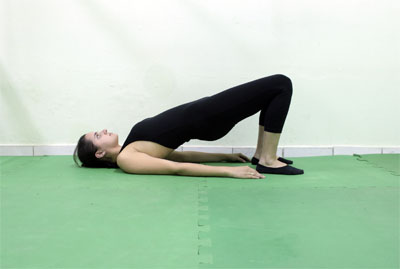 |
| **Roll over** | Patient in supine with arms along the body  Patient should lift both lower limbs up to 90 degrees of hip flexion.  Repeat 4-10 times.  Return to the starting position. | 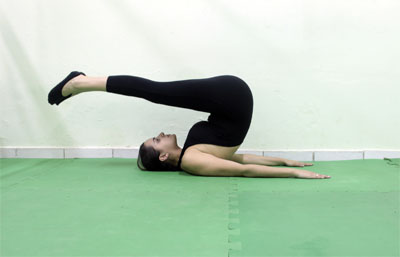 |
| **One Leg Kick** | Patient in prone supported by the forearms (sphinx position).  Patient flexes one of the knees up to 90 degrees and then kicks backwards (twice), alternating the legs.  Repeat 4-10 times.  Return to the starting position. | 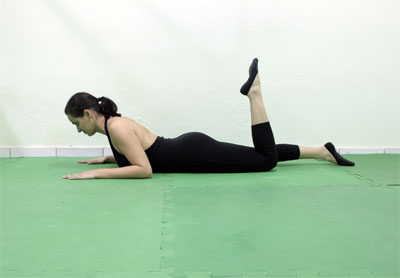 |
| **The Saw** | Patient sits with both legs and arms abducted.  Patient flexes and rotates the trunk moving one hand towards the contralateral foot, alternating the movements.  Repeat 4-10 times.  Return to the starting position. | 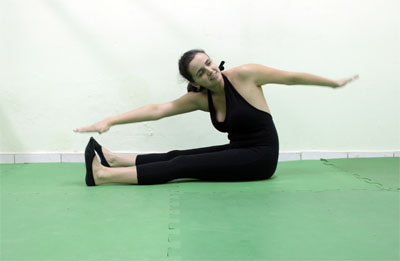 |
| **Side Kick** | Patient in lateral decubitus with the body aligned. The head is supported by on the hands while the other hand touches the mat.  Patient kicks with one leg using all range of motion backwards and forwards. Alternating the sides.  Repeat 4-10 times.  Return to the starting position. | 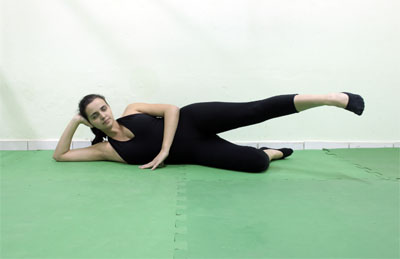 |
| **Side Leg Lifts** | Patient in lateral decubitus with the body aligned. The head is supported by on the hand while the other hand touches the mat.  Patient lifts both legs.  Repeat 4-10 times.  Return to the starting position. | 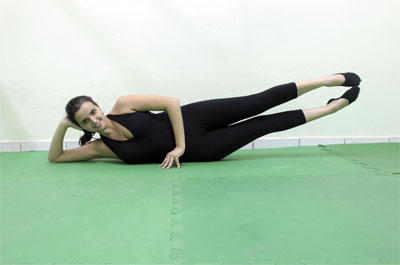 |
| **Double Leg Kick** | Patient in prone position with both legs close to each other and hand behind the back.  Patient extends the trunk and flexes both lower limbs simultaneously.  Repeat 4-10 times.  Return to the starting position. | 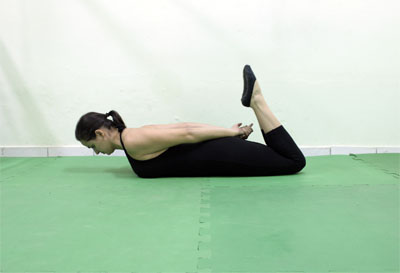 |
| **Spine Stretch** | Patient sits with his/her legs straight and apart from each other.  Patient move his/her hands towards the feet making a C-curve.  Repeat 4-10 times.  Return to the starting position. | 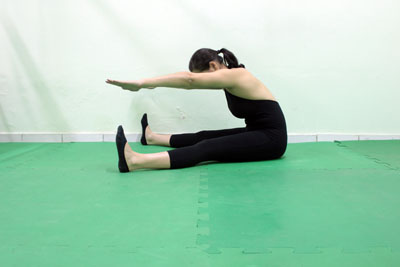 |
| **Swimming** | Patient in prone position with lower limbs close to each other with his/her upper limbs fully extended.  Patient extends one upper limb and the contralateral lower limb simultaneously. Then patient starts simulating a swimming (crawl stroke).  Repeat 4-10 times.  Return to the starting position. | 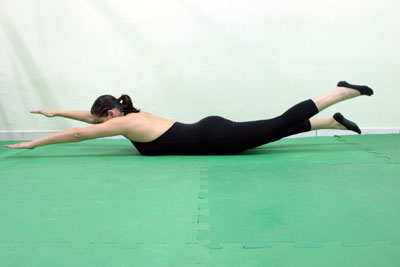 |
| **Mermaid Side Stretch** | Patient sits with both lower limbs flexed with a side support to one side only.  The opposite hand touches the mat in order to provide balance and support.  The free hand moves above the head bending the trunk towards to hand that is touching the mat, then change sides.  Repeat 4-10 times.  Return to the starting position. | 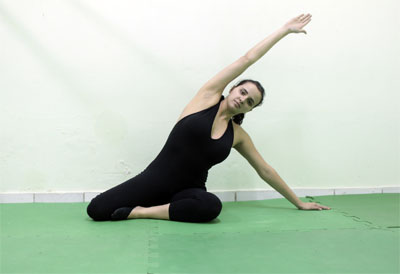 |
